# Neighbourhood Continuity Is Not Required for Correct Testis Gene Expression in *Drosophila*

**DOI:** 10.1371/journal.pbio.1000552

**Published:** 2010-11-30

**Authors:** Lisa A. Meadows, Yuk Sang Chan, John Roote, Steven Russell

**Affiliations:** 1 Department of Genetics, University of Cambridge, Cambridge, United Kingdom; 2 Cambridge Systems Biology Centre, University of Cambridge, Cambridge, United Kingdom; University of California Berkeley/Howard Hughes Medical Institute, United States of America

## Abstract

It is now widely accepted that gene organisation in eukaryotic genomes is non-random and it is proposed that such organisation may be important for gene expression and genome evolution. In particular, the results of several large-scale gene expression analyses in a range of organisms from yeast to human indicate that sets of genes with similar tissue-specific or temporal expression profiles are clustered within the genome in gene expression neighbourhoods. While the existence of neighbourhoods is clearly established, the underlying reason for this facet of genome organisation is currently unclear and there is little experimental evidence that addresses the genomic requisites for neighbourhood organisation. We report the targeted disruption of three well-defined male-specific gene expression neighbourhoods in the *Drosophila* genome by the synthesis of precisely mapped chromosomal inversions. We compare gene expression in individuals carrying inverted chromosomes with their non-inverted but otherwise identical progenitors using whole-transcriptome microarray analysis, validating these data with specific quantitative real-time PCR assays. For each neighbourhood we generate and examine multiple inversions. We find no significant differences in the expression of genes that define each of the neighbourhoods. We further show that the inversions spatially separate both halves of a neighbourhood in the nucleus. Thus, models explaining neighbourhood organisation in terms of local sequence interactions, enhancer crosstalk, or short-range chromatin effects are unlikely to account for this facet of genome organisation. Our study challenges the notion that, at least in the case of the testis, expression neighbourhoods are a feature of eukaryotic genome organisation necessary for correct gene expression.

## Abstract 2

Disrupting the linear organization of testis gene expression neighborhoods in the *Drosophila* genome does not affect gene expression, suggesting that neighborhood organization is not primarily driven by gene expression requirements.

## Author Summary

The order of genes within eukaryotic genomes is not completely random. In all genomes characterised to date there are regions of the genome, known as gene expression neighbourhoods, which contain clusters of genes that are expressed together in a particular tissue or at a particular developmental stage. Comparative genomics indicates that at least some neighbourhoods have been conserved during evolution, suggesting that this facet of genome organisation may be functionally advantageous. While several models explaining the organisation of the genome into neighbourhoods have been proposed, the functional significance of neighbourhood organisation has not been experimentally tested. Here, we report experiments that disrupt defined testis gene expression neighbourhoods in the *Drosophila* genome. We generated chromosomal inversions with a breakpoint within a neighbourhood, defined as having genes co-expressed within the testis. Comparing gene expression in flies carrying inversions with their otherwise identical progenitors shows that maintaining the linear organisation of genes in a neighbourhood is not necessary for correct gene expression. We also show that it is not necessary for genes in a neighbourhood to be in close proximity in the nucleus for them to be co-expressed, since the inversions disrupt the spatial organisation of neighbourhood genes in the nucleus. Our experiments indicate that the current models used to account for the existence of gene expression neighbourhoods are unlikely to be sufficient.

## Introduction

Understanding gene regulation and genome organisation presents a complex challenge. Traditional techniques typically involve a gene-by-gene approach and provide a wealth of information about the control regions at which transcription factors and repressors bind to regulate transcription. The more recent use of genome-wide approaches enables the expression levels of all genes in a genome to be analysed simultaneously and the increasing collections of such data has led to the idea that genes are not only controlled individually but may also be regulated according to their location in the genome. The idea that genomic location has an impact on gene regulation is not new, since it is well established from work in several species that the expression pattern or activity of transgenes is influenced by genomic insertion site [1]–[3]. There is accumulating evidence from statistical analyses of genome-wide expression data, derived from both microarray and sequencing-based assays, that suggests gene order in eukaryotic genomes is not random and that genes with similar expression profiles tend to be clustered within genomic neighbourhoods. Genome-scale studies with the budding yeast *Saccharomyces cerevisiae* were the first to indicate clustering of coexpressed genes [4]–[6]. Subsequently, this phenomenon of non-random clustering of similarly expressed genes in localised genomic neighbourhoods has been observed in all metazoan organisms examined, including *Arabidopsis thaliana* [7],[8], *Caenorhabditis elegans* [9]–[12], *Drosophila melanogaster* [13]–[16], mouse [17],[18], and humans [19]–[21].

While clustering can be partially accounted for by features such as overlapping genes, tandemly duplicated genes, homologous genes, and operons (for example, in *C. elegans* the coexpression of neighbouring genes is mostly due to operons and duplicate genes [11]), the majority of co-expression neighbourhoods cannot be accounted for in these ways. The analysis of several species has shown that there is a significant tendency for genes in the same metabolic pathway to cluster, although the patterns of pathway clustering appear to be species-specific [22]. In the human genome there is a general trend for clustering of genes that are expressed across most tissues (housekeeping genes), whereas clustering of genes expressed in specific tissues is less apparent [20]. In *Drosophila melanogaster*, clustering of testis-specific genes is well described: one analysis of EST expression [13] showed that approximately 45% of genes uniquely expressed in the testes cluster in neighbourhoods of at least four contiguous genes. An analysis based on a series of microarray studies allowed for clusters that contain intervening genes with different expression patterns and concluded that more than 20% of genes in the *Drosophila* genome are clustered into neighbourhoods [14]. The study identified approximately 200 neighbourhoods of 20 to 200 kb across the fly genome, each containing 10–30 adjacent co-regulated genes. The genes defining each neighbourhood are not functionally related in any obvious way, although some of the neighbourhoods represent genes with testis-enriched expression. A more stringent statistical analysis of sex-specific gene expression identified a smaller number of neighbourhoods associated with testis expression [23]; some of these correspond to the testis neighbourhoods from the large microarray study [14]. Clustering of testis expressed genes is not unique to *Drosophila*, with testis expression neighbourhoods also identified in the mouse [17],[18].

Genes displaying marked sexually dimorphic expression are under different evolutionary constraints than genes expressed equally between the sexes; for example, male-biased genes are under-represented on the *X-*chromosome and show greater sequence divergence compared to female biased genes [24],[25] and a variety of studies have identified considerable variation in expression levels of male-specific genes both within and between species [26]–[28]. While it is possible that there may be different mechanisms acting to select testis neighbourhoods compared to gene expression neighbourhoods in somatic tissues, there is no *a priori* reason to indicate this.

Although there is extensive evidence indicating that co-expressed genes cluster into neighbourhoods across all major eukaryotic phyla, the mechanism(s) behind this organisation and the functional significance of gene co-expression neighbourhoods is currently unclear. If co-expression neighbourhoods are non-functional and/or purely coincidental, we would not expect them to be conserved during evolution. In contrast, a comparison between *S. cerevisiae* and *C. albicans* indicates that co-expressed genes are conserved more than expected by chance [29]. An analysis of metazoan genomes indicates natural chromosomal breakpoints tend to avoid gene expression neighbourhoods; for example, breakpoints within neighbourhoods are under-represented when comparing *Drosophila* species [30], human and mouse [31],[32], or human and chicken genomes [33]. Finally, the majority of neighbourhoods defined in the *D. melanogaster* genome have been conserved across the 12 sequenced *Drosophila* species [16],[34],[35]. Taken together, these data support the view that at least some neighbourhoods are functionally advantageous and thus conserved by natural selection.

A variety of models have been proposed to explain the existence of gene expression neighbourhoods, including (i) those invoking the local activity of transcription factors via one or more closely located regulatory sequences, (ii) models that suppose coordinate regulation through local structural features of chromatin organisation such as boundary elements, and (iii) long-range effects due to higher order aspects of chromatin organisation in the nucleus [36]–[40]. While each of these models has attractive features, there is little experimental evidence available to evaluate the likely contribution of each of these effects. Thus despite a plethora of reports describing the existence of neighbourhoods, experiments formally testing the requirement for this aspect of genome organisation for normal gene expression are currently lacking. Here we address this issue by generating precisely mapped chromosomal inversions that target the disruption of testis gene expression neighbourhoods in the *Drosophila* genome. Using microarray analysis to compare gene expression in individuals carrying inverted chromosomes with their non-inverted but otherwise identical progenitors, we find there are no significant differences in the expression of genes that define the neighbourhoods. Our experiments indicate that in the fly testis the organisation of genes into expression neighbourhood clusters is not essential for their normal expression.

## Results

**Figure 1:**
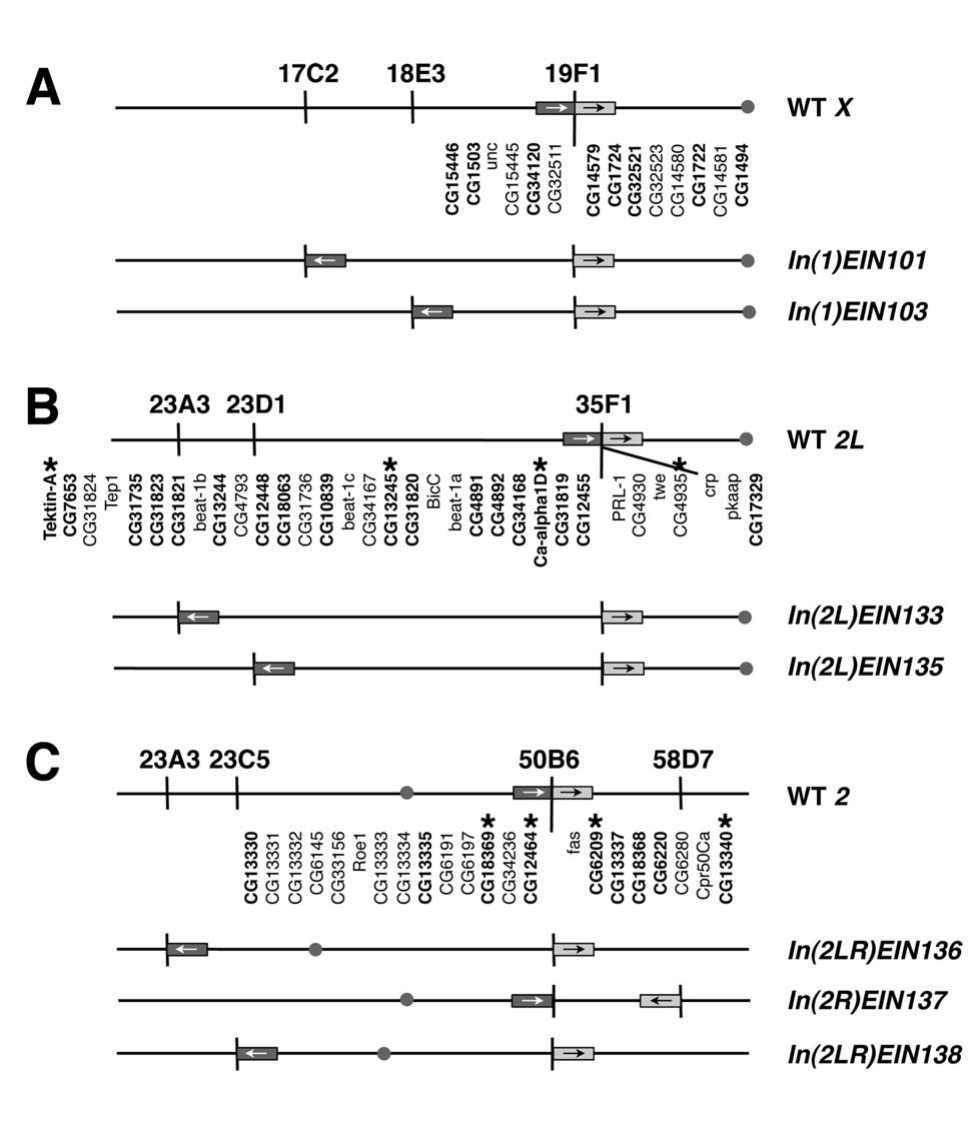
Inversions disrupting male-specific gene expression neighbourhoods. Diagrammatic representation of the seven inversions generated in three expression neighbourhoods. (A) Inversions in the *X* chromosome neighbourhood at 19F. (B) The Chromosome *2L* inversions disrupt the 35F neighbourhood. (C) Inversions disrupting the chromosome *3R* neighbourhood at 50B. Each of the three neighbourhoods is represented by the shaded boxes with the arrows indicating relative orientation. The genes in the neighbourhood are indicated underneath with bold text indicating male elevated expression; the gap represents the location of the breakpoint in each neighbourhood. Note that the inversions in the 35F region have two different breakpoints. The chromosomal location of each breakpoint is indicated below the wild type chromosome cartoon. Below the wild type, the structure of each inversion is diagrammed to show how the relative position and orientation of genes in the neighbourhood changes. The grey circles represent the centromeres and the stars represent genes assayed by RT-PCR.

If the genes within a neighbourhood need to be contiguous for their observed co-expression, then altering their proximity should result in changes in gene expression. However, if the linear association of genes in a neighbourhood is not essential for co-expression, then disrupting neighbourhoods will have little impact on gene expression. To examine these alternatives we tested the effect of disrupting the continuity of a set of neighbourhoods in the *Drosophila* genome by generating chromosomal inversions with one breakpoint within a neighbourhood and a second breakpoint some distance away. We chose a set of three well-defined gene expression neighbourhoods associated with male-specific expression [13],[14],[23] and used an FRT-based recombination system to induce precisely defined chromosomal inversions with breakpoints within each neighbourhood (Figure 1). We compared gene expression in individuals carrying the inversion with individuals carrying un-inverted chromosomes, but that are otherwise genetically identical. The relevant chromosomes of the latter individuals harbour the two FRT-bearing RS elements that direct the recombination event and are referred to as *cis* stocks (see below).

Two of the neighbourhoods (35F and 50B) were identified by two independent microarray studies and an EST analysis [13],[14],[23]. Although the third neighbourhood (19F) was not identified by the stringent statistical threshold used in one of the microarray studies [23], it was selected for analysis since the *X* chromosome is known to be underrepresented for testis-expressed genes in *Drosophila* [24] and may be under different evolutionary constraints in terms of genome organisation. Since sex-specifically expressed genes are known to vary considerably between different *Drosophila* strains [26],[27],[41], we compared male and female gene expression in one of our inversion stocks and in its un-inverted progenitor (the *cis* stock) to confirm male-specific expression in the selected neighbourhoods. This analysis demonstrates that the microarrays we use are reproducible when inversions and progenitor stocks are compared since the male-female expression ratios are similar across both experiments (Table S1). We also examined the FlyAtlas tissue expression database [42] to confirm the male-specific expression profile of the genes within each neighbourhood (Table S1). These data demonstrate that the selected neighbourhoods are over-represented for genes that are predominantly expressed in the testis.

Inversions were constructed using RS3 and RS5 *P* elements generated by the DrosDel project (Figure S1) [43],[44]. Importantly, the only difference between the non-inverted and inverted chromosomes is that the latter carries a functional copy of the *white* reporter gene while the former carries the separate 5′ and 3′ ends of the gene; otherwise the genetic background of inversion and *cis*-stocks are identical. We generated seven inversions, six of which are homozygous viable and fertile (Table 1), and verified them by genomic PCR and polytene chromosome cytology (Figure S2). For gene expression analysis we used oligonucleotide microarrays to directly compare RNA from inversion stocks with their un-inverted progenitors, biologically replicating each comparison at least three times but usually four. A summary of the expression data for genes in each neighbourhood is provided in Table S2 with the full dataset in Table S3.

**Table 1:** Inversions generated.

Domain	Size	Elevated	Inversion	Element 1	Location	Element 2	Location	Cytology
19F	190 kb	8 (14)	*In(1)EIN101*	CB-6796-3	21042575	5-SZ-4085	18428636	17C; 19F
			*In(1)EIN103*	CB-6796-3	21042575	5-SZ-3054	19607527	18E: 19F
								
35F	238 kb	18 (33)	*In(2L)EIN133*	5-SZ-4036	16251443	CB-5496-3	2750975	23A; 35F
			*In(2L)EIN135*	CB-5425-3	16281817	5-HA-2004	3055770	23D; 35F
								
50B	265 kb	9 (22)	*In(2LR)EIN136*	CB-5832-3	9510372	5-SZ-3330	2750920	23A; 50B
			*In(2R)EIN137*	CB-5832-3	9510372	5-HA-1133	18289458	50B; 58D
			*In(2LR)EIN138*	5-SZ-3339	9510512	CB-0114-3	3018404	23C; 50B
								
18E	160 kb	11 (17)	*In(1)EIN103*	CB-6796-3	21042575	5-SZ-3054	19607527	18E; 19F

Domain  =  gene expression neighbourhood cytological region according to the standard *Drosophila* cytological map. Size  =  approximate neighbourhood size. Elevated  =  number of male elevated genes or embryo elevated in the 18E domain (total number of genes). Each inversion along with the starting RS elements and their genomic locations are indicated. Cytology  =  location of the second breakpoint on the cytological map.

**Figure 2:**
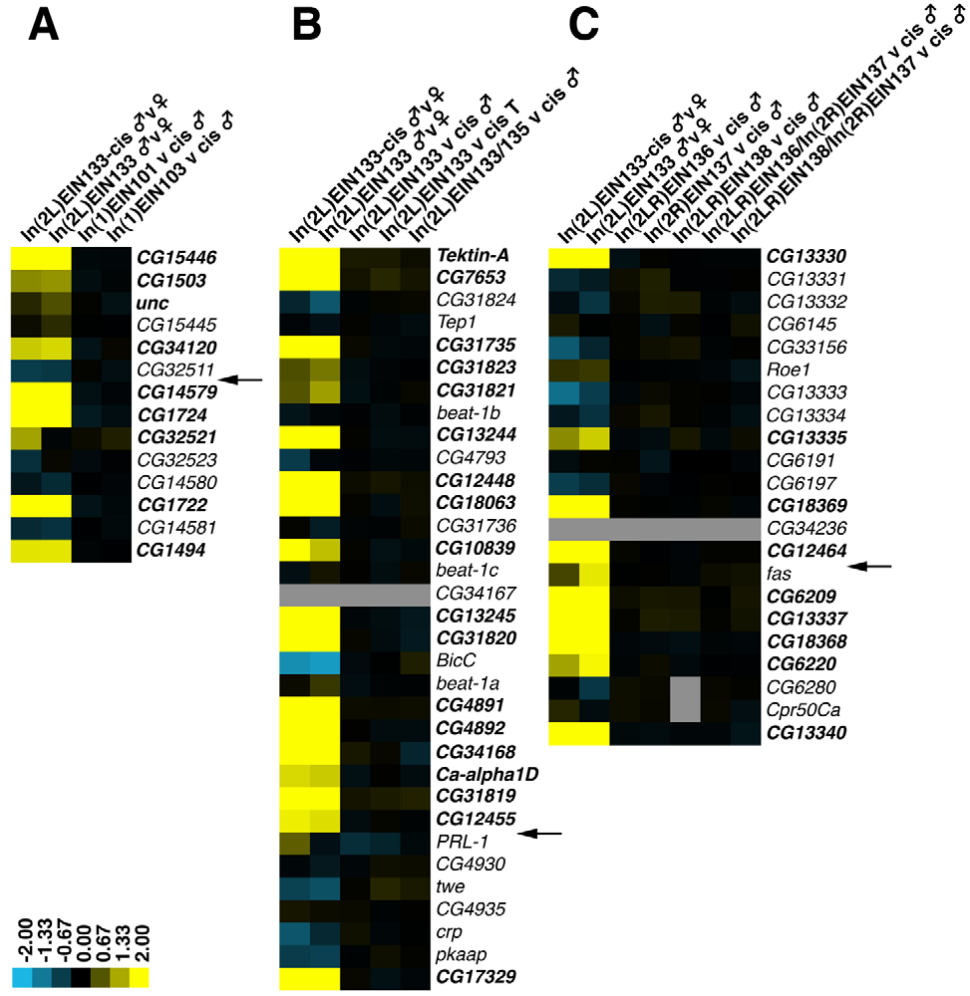
Microarray analysis of gene expression. (A) 19F, (B) 35F, and (C) 50B. For each neighbourhood the heatmaps display the average log2 expression ratio of each gene in the indicated comparisons according to the colour scale at the bottom. The location of the inversion breakpoint within each neighbourhood is indicated by the arrows. Data from the *In(2L)EIN133* inversion and cis male versus female comparisons are shown for each neighbourhood. The “*cis*” designation is shorthand for the uninverted progenitor chromosome that carries the 2 *RS* insertions used to direct the recombination event. Grey regions indicate no data. While the male versus female comparisons show strong male biased expression (yellow), when inversion males are compared to their non-inversion progenitors there is very little change in expression (black). T, testis.

For the *X* chromosome we created two inversions disrupting a 190 kb neighbourhood at 19F [14]. Both inversions break the neighbourhood between *CG32511* and *CG14579*, with their other breakpoints at 17C (*In(1)EIN101*) and 18E (*In(1)EIN103*), respectively (Figure 1A). A comparison of males carrying the inverted *X* chromosomes with males carrying the un-inverted progenitor chromosomes (*cis* stocks) showed very minor changes in gene expression levels (Figure 2A). Three genes (*CG14579*, *CG1724*, and *CG1722*) showed 1.2- to 1.3-fold down regulation in *In(1)EIN101* males (*p*<0.05), but of these only *CG1722* shows a change in *In(1)EIN103* males (1.1-fold). At the other breakpoints of the inversions there were no significant changes in gene expression between inverted and un-inverted stocks (Figure S3). Importantly, we find that there is no difference in the signal to noise ratios of intensity (A) values or in the 95% confidence limit range for the ratio (M) values when we compare the measurements observed in inverted neighbourhoods with those experiments in which the neighbourhood is not inverted (Figures S7 and S8; Tables S8 and S9). This supports the view that the inversions do not significantly alter the expression of the genes within the neighbourhood.

The *X-*chromosome inversions are relatively small, encompassing 1.4–2.6 Mb of chromatin, and it is possible that local chromatin effects may still be active over this distance. To explore this we disrupted a neighbourhood at 35F with larger inversions, involving approximately 13 Mb of chromosome arm *2L*. We made two inversions starting from slightly different places at the distal end of the neighbourhood (Figure 1B). One inversion, *In(2L)EIN133*, breaks within an intron of the *PRL-1* gene but has no apparent phenotype. Homozygous *In(2L)EIN133* males show a slight but significant reduction in *PRL-1* expression (1.7-fold, *p*<0.05), but otherwise there are no significant changes in expression compared to the *cis-*progenitor. The reduction in *PRL-1* expression is most likely a direct consequence of disrupting *PRL-1* regulatory sequences since there is no change in expression observed with the second inversion (Figure 2B). We considered the possibility that using RNA from whole males might obscure small changes in gene expression in the testis and therefore compared expression in the dissected testes of inversion and non-inversion males. We see a slight change in *PRL-1* expression, but otherwise the genes are similarly expressed in the inversion and *cis*-stocks (Figure 2B). The second inversion, *In(2L)EIN135*, is homozygous lethal due to disruption of *cropped* (*crp*), and we therefore generated males transheterozygous for *In(2L)EIN133* and *In(2L)EIN135*, which we compared with males transheterozygous for the respective *cis*-stocks. Again we see very few significant changes in the expression of neighbourhood genes in the transheterozygotes apart from a 1.5-fold reduction in CG34168. This gene does not change expression in the *In(2L)EIN133* homozygotes, suggesting it is a local effect from the 23D end of *In(2L)EIN135*. At the 23A end of *In(2L)EIN133* we see a slight increase in *Pgk* expression in testis, suggesting it may be influenced by the male-specific expression of the neighbourhood gene *CG12455* (Figure S4).

To disrupt genome organisation to a greater extent we generated paracentric (*In(2R)EIN137*) and pericentric (*In(2LR)EIN136* and *In(2LR)EIN138*) inversions interrupting a neighbourhood at 50B on chromosome arm *2R* (Figure 1C). The latter two inversions involve over 30 Mb of euchromatin along with the centromeric heterochromatin and encompass approximately 25% of the euchromatic genome. As before, inversion-bearing males were compared to their un-inverted progenitors and showed no significant impact on gene expression (Figure 2C). To try and eliminate any contributions that chromosome pairing effects may make to gene expression in the neighbourhood, we generated transheterozygotes between the pericentric inversions and the *In(2R)EIN137* paracentric inversion. In this case we expect the somatic pairing of homologous chromosomes to be completely disrupted [45]. Again we found no significant differences in expression between inverted and non-inverted lines (Figure S5).

**Figure 3:**
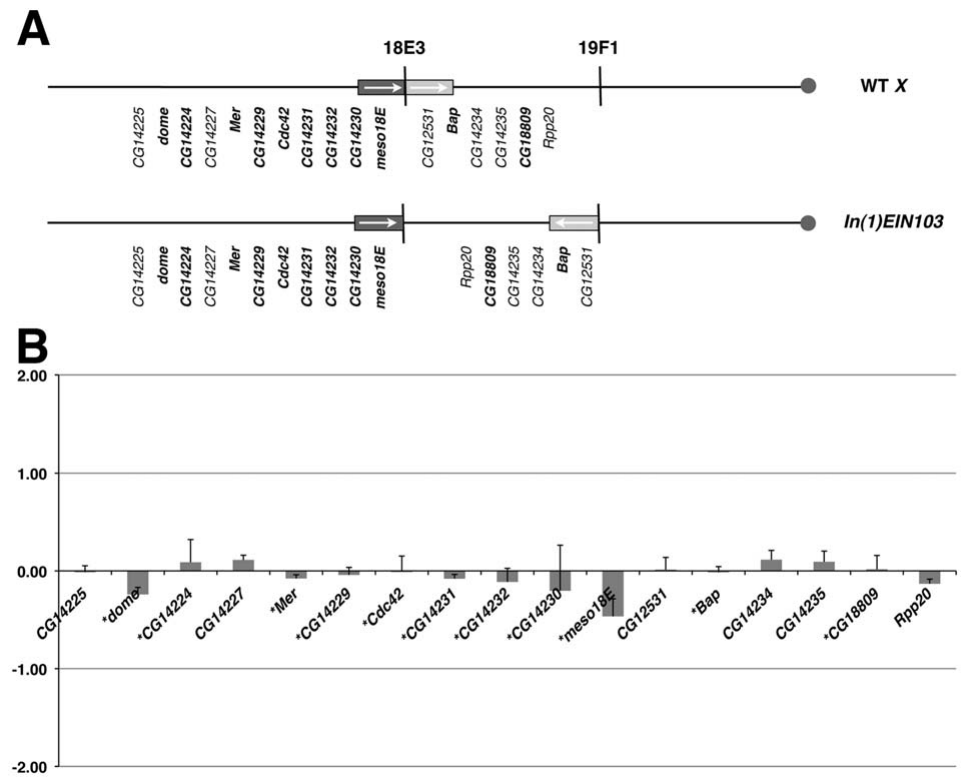
Disrupting an embryo gene expression neighbourhood. (A) *In(1)EIN103* disrupts an embryo neighbourhood at 18E; the genes in the neighbourhood and their location in the wild type and inverted chromosomes is indicated. (B) Graph of gene expression ratios from a microarray comparison of *In(1)EIN103* and its uninverted progenitor. The *y*-axis represents log2 expression ratios.

Clustering of testis-specific genes is not unique to *Drosophila*, since it has also been shown that a large proportion of testis-specific genes are similarly clustered in mouse [18]. However, it may be argued that the testis represents a highly derived specialised organ dedicated to spermatogenesis and it is possible that the lack of significant gene expression effects in the inversion stocks may not be representative of other tissues, especially if they are under different evolutionary constraints. To begin to address this issue we investigated the effect of disrupting a 60 kb embryo-specific neighbourhood between CG14255 and CG32530 at 18E3 (Spellman block 209, [14] Figure S6) with the other breakpoint at 19F (*In(1)EIN103*, Table 1). Once again we observed no significant gene expression differences in the genes defining the neighbourhood when inversion and *cis* stocks are compared (Figure 3). A summary of the expression data for genes in this neighbourhood is provided in Table S2 and the full genome dataset in Table S4.

**Figure 4:**
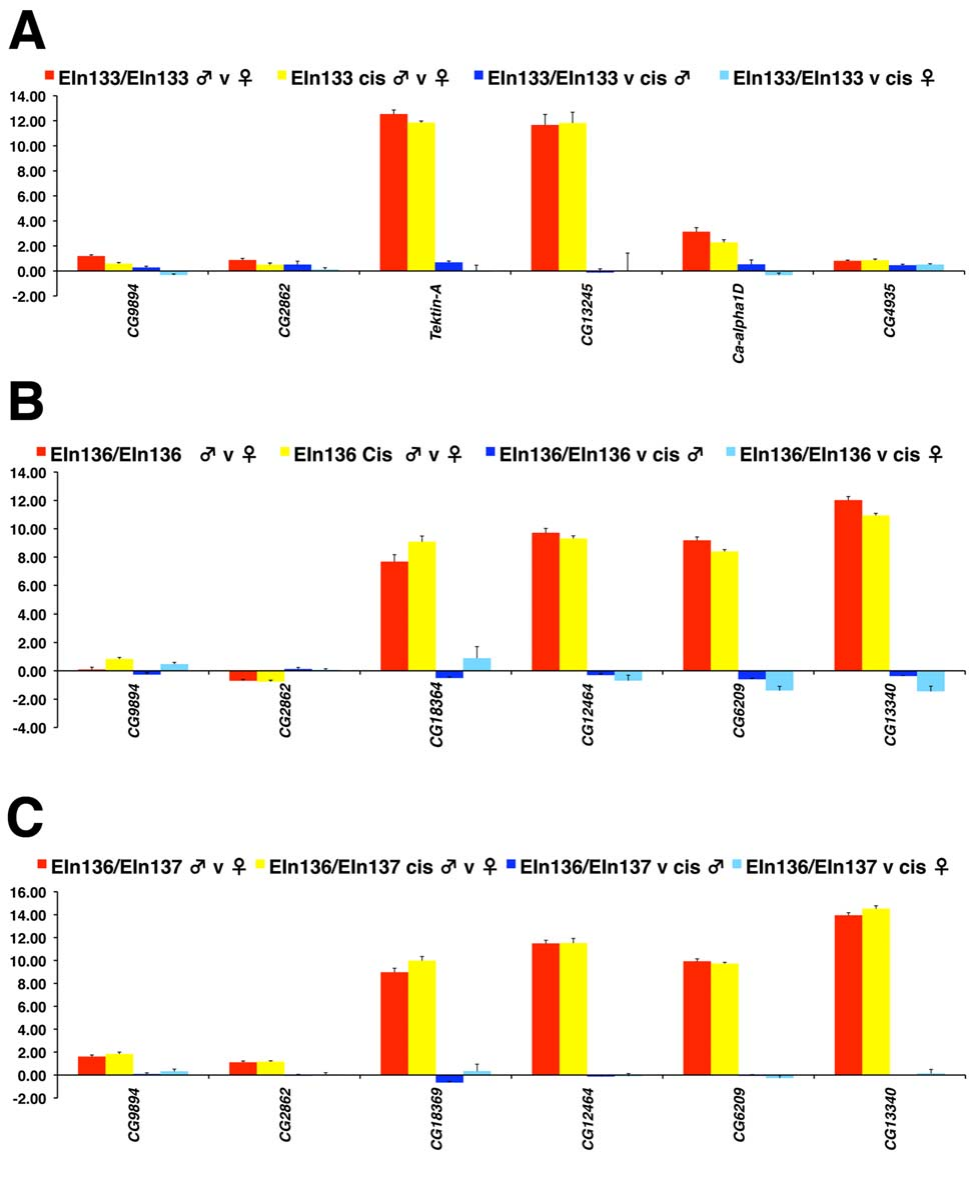
Confirmation by quantitative Real-Time PCR. (A) Supporting the microarray experiments, four genes in the 35F neighbourhood and two genes flanking the other end of the inversion breakpoint at 23A3 show expected male-specific expression and no change in expression when inversion bearing males are compared with undisrupted progenitors. (B and C) Similar confirmation of the microarray data is seen with homozygous (B) and transheterozygous (C) inversions in the 50B neighbourhood. Note the small change in expression detected in females in (B).

While the microarray platform we use has been shown to perform well in detecting gene expression changes [46], we elected to validate our microarray data by quantitative Reverse Transcription PCR. We selected seven male-biased and three control genes, testing gene expression in males and females from three different inversion stocks and their *cis* progenitors. The gene expression estimates from quadruplicated qRT-PCR assays are very similar to those obtained with the microarray analysis and again show no significant changes in male-specific gene expression associated with the inversion (Figure 4). We do however observe slight expression changes in females homozygous for *In(2LR)EIN136*, indicating the assay is sensitive to small changes in expression.

While there are no changes in the expression of neighbourhood genes in the inversion stocks, we do see effects on the expression of genes elsewhere in the genome in some stocks (Table S5). In general there are very few effects on gene expression with the chromosome 2 inversions: between 11 and 57 genes across the entire genome with significant changes (1.5-fold: *p*<0.01) in the testis or whole males. In the case of the *X* chromosome inversions we found that 176 (*In(1)EIN101*) and 138 (*In(1)EIN103*) genes showed significant changes in whole adult males. However, the vast majority of the affected genes encode proteolytic functions associated with the midgut (*p* = 4.1E-09) and are likely to reflect environmental or gut flora differences introduced by the crossing scheme used to generate the *X* inversions. In support of this we find that these gene expression changes are not seen in the analysis of *In(1)EIN103* in embryos, where we only detect 13 genes across the whole genome with significant expression changes (1.5-fold: *p*<0.01, Table S5).

**Figure 5:**
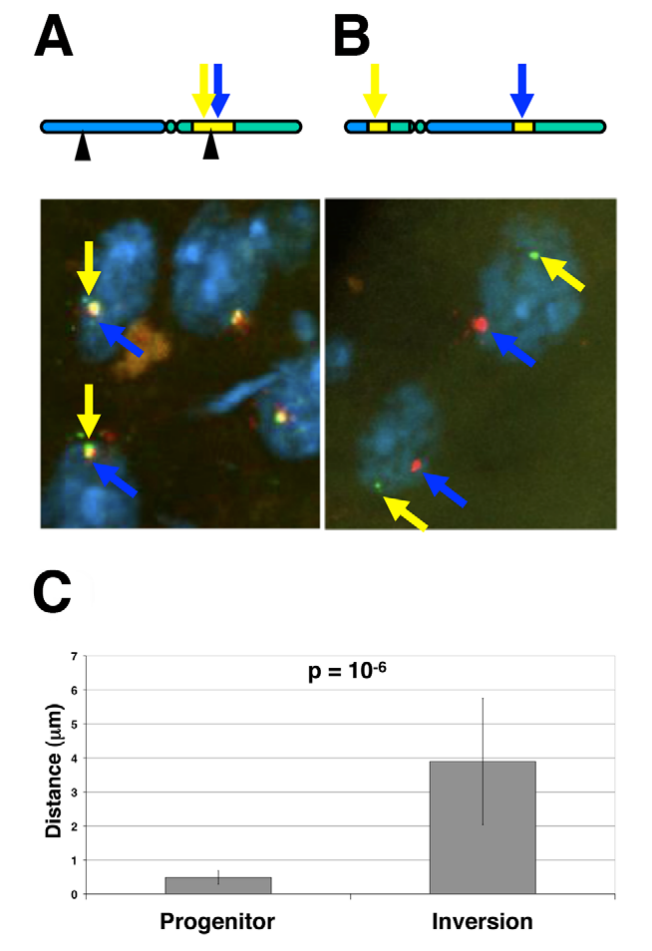
Two colour DNA FISH in spermatocytes . Confocal microscopy and 3D measurement of separation distances between proximal and distal probes. DNA FISH 3D reconstructed image from confocal stack on spermatocytes of (A) progenitor stock and (B) inverted stock. Proximal probe (A555, yellow arrow), distal probe (A488, blue arrow). Nuclei are counterstained with DAPI. (C) Histogram plot of separation distances between proximal and distal probes in progenitor (*n* = 29) and inversion (*n* = 17).

We have engineered inversions to separate two halves of gene expression neighbourhoods by genomic distances of up to 30 Mb. While the inversions certainly disrupt the linear organisation of the chromosome, it is possible that the two distant regions of the inversion can re-associate in the nucleus and come into close proximity in the same sub-nuclear compartment. While we argue this is unlikely to be the case where we have disrupted somatic pairing of homologous chromosomes by combining para- and peri-centric inversions, we cannot eliminate the possibility. The association of neighbourhoods in the three-dimensional space of the nucleus offers a plausible explanation as to why neighbourhood gene expression is unaffected by the inversions. To test this possibility we employed two colour DNA fluorescence *in situ* hybridisation (DNA FISH) to measure the distance between probes that recognise DNA sequences flanking the *In(2LR)EIN136* inversion breakpoint disrupting the neighbourhood at 50B. We hybridised the fluorescent probes to dissected testes from inversion homozygotes and measured the distance between the two different fluorescent signals in spermatocyte nuclei. Linearly, the two fluorescent probes are separated by a genomic distance of less than 25 kb in the un-inverted neighbourhood and greater than 30 Mb after inversion. If the two halves of the disrupted neighbourhood come together in three-dimensional space, then we expect the distance between signals from the two probes to be close together in the spermatocyte nucleus. However, our measurements (Figure 5) clearly show that there is a significant difference between the probe distances in the inversion (mean  = 3.89 microns, SD = 1.86, *n* = 17) compared to the un-inverted progenitor (mean  = 0.48 microns, SD = 0.19, *n* = 29) (*p* = 10^−6^). Combined with the gene expression data, our analysis clearly indicates that although genes in the two separated parts of the inverted neighbourhood are in distant territories within the spermatocyte nucleus, they are nevertheless expressed at levels similar to those in their native un-inverted configuration. Co-localisation of genes in a neighbourhood to the same physical region of nucleus is therefore unlikely to be a critical mechanism for their co-expression.

## Discussion

Regulation of gene expression is subject to multiple layers of control [47],[48]. While the expression of an individual gene is generally independently controlled by its promoter and associated regulatory elements, it may also be regulated by local epigenetic mechanisms such as DNA methylation, histone modification, and chromatin remodelling [49]. The discovery of clusters of co-expressed genes in many organisms has lead to the suggestion that gene expression is additionally regulated by genome position. Non-random organisation of the genome allows compartmentalisation of the nuclear space: at a simple level this could be separating active and inactive genes. Such organisation may help enhance the efficiency of transcriptional activation or repression and evidence is growing that there is indeed a spatial component to gene regulation and genome evolution (reviewed in [50]–[52]). One facet of gene organisation is suggested to be the clustering of genes into expression neighbourhoods. In this study we conclude, at least for the well-defined gene expression neighbourhoods we have examined, that the contiguous physical organisation of genes in neighbourhoods is not necessary for the correct expression of the genes defining that neighbourhood. It is possible that disrupting gene expression neighbourhoods results in changes in gene expression levels that are too subtle to be detected by the microarray or PCR assays we used. While we recognise this we note that we are able to reproducibly detect small changes in expression (1.2-fold), and therefore we are confident in asserting that neighbourhood organisation is unlikely to be a major contributor to gene expression.

A second caveat is that our inversions rely on transposable elements carrying recombination sites and it is possible that the element we use inserts non-randomly with respect to gene expression neighbourhoods. For example, with the *X* chromosome inversion, the RS element is inserted in a 30 kb region between divergent genes and it is conceivable that this may be a natural break separating two smaller but independent neighbourhoods. While the analysis of three independent neighbourhoods suggests that it is unlikely that this occurs in all three cases, we must nevertheless consider this a possibility and we are currently disrupting other neighbourhoods to confirm or refute our conclusions.

Several methods have been proposed to account for gene expression neighbourhoods, including bystander gene activation or the ripple effect, whereby genes are activated simply because of their proximity to another intensively transcribed gene [53],[54]. Other models invoke local effects from strong enhancers, co-regulating all of the genes in a neighbourhood [40] or unique local chromatin domains [55]. Based on our disruption experiments, these models are unlikely to account for gene expression neighbourhoods since the inversions remove at least some of the neighbourhood genes away from any local enhancers as well as disrupting putative chromatin domains.

Interestingly, a recent analysis of gene expression in males of seven *Drosophila* species indicates that two of the neighbourhoods we examined (19F and 50B) are conserved co-expression domains while the third (35F) lies adjacent to a conserved neighbourhood [16]. It has been proposed that at least some gene expression neighbourhoods are conserved in related species [34],[56], including mammals [31],[32]. Our analysis suggests that such evolutionary conservation is not driven by selection for *cis*-acting regulatory influences.

Higher order features of genome architecture have been proposed to account for co-expression of neighbouring genes [37],[39],[57]. The coupling of gene co-expression in neighbourhoods could be controlled by particular histone modifications, initiated at specific sites and spreading along a chromosomal region until a boundary element such as an insulator is reached (reviewed in [58]–[60]). This type of chromatin domain organisation may explain the existence of some neighbourhoods, however there is little association between the currently mapped insulator-binding proteins and the boundaries of expression neighbourhoods in the *Drosophila* embryo [61]. Therefore, although we cannot rule out the possibility that insulators or other, as yet unknown, sequence or protein features define the boundaries of gene expression neighbourhoods, we can conclude that separating boundaries has no obvious effect on gene expression.

We explored the possibility that despite disrupting linear chromosome organisation the inversions do not affect three-dimensional organisation of chromatin in the nucleus. First we generated transheterozygotes with a peri- and paracentric inversion to severely disrupt chromosome organisation, and second we directly assayed nuclear location by a fluorescent *in situ* hybridisation assay. We show that the two halves of a neighbourhood which are separated by an inversion do not associate within the same sub-nuclear territory. We therefore conclude that it is not essential for neighbourhood genes to be in close proximity in the spermatocyte nucleus for normal levels of gene expression. Of course we cannot rule out that association between the separated parts of the neighbourhood may occur transiently, for example during the initiation of gene expression, and that such dynamic interactions may not be captured by our DNA FISH analysis. The eukaryotic interphase nucleus is known to be a highly compartmentalised, organised, and dynamic organelle (reviewed in [62],[63]). There have been several examples demonstrating how the activity of genes is linked to their position within the nucleus ([64]–[66]; reviewed in [67]), and it is likely that sub-nuclear positioning contributes to optimising gene activity. Some distant genes associate via chromatin looping to specific regions of the nucleus containing high local concentrations of transcriptional and mRNA-processing machinery, known as transcription factories [68],[69]. Presumably this organisation contributes to more effective coordination of transcription, although the functional significance of transcription factories is currently unclear. If the nuclear concentration of a transcription factor is limiting [70],[71], localising genes regulated by similar factors could potentially lead to more efficient co-regulation, however loss of this localisation may not necessarily be deleterious. We therefore speculate that gene expression neighbourhoods have tended to remain intact during evolution due to the likelihood that the genes would be more efficiently co-transcribed. However, it is clear from our neighbourhood disruption experiments that co-expression is not absolutely dependent on close gene proximity.

While we examined a somatic gene expression neighbourhood and found no expression changes associated with inversion, the majority of our experiments were carried out with testis neighbourhoods and we cannot eliminate the possibility that the spermatocyte nucleus represents a special case of neighbourhood organisation. For example, it is known that the dramatic changes in gene expression characterising the spermatogenic programme are associated with the deployment of a specific set of basal transcription factors, the testis-specific TAFs, and the reorganisation of at least some aspects of chromatin structure [72]–[74]. Similar types of changes are also associated with mammalian spermatogenesis [75],. Thus it is possible that in the testis, neighbourhoods have arisen because they facilitate the organisation of TAFs into the type of transcription factories described above. In this scenario we imagine that testis expression is slightly more efficient with neighbourhood organisation but that the small effects resulting from simply dividing the neighbourhood in two are not visible at the level of resolution we can achieve. Since our current understanding of testis gene expression indicates each gene is likely to be regulated by discrete, specialised testis promoters [74], it may be that a single breakpoint has only a very small effect. Clearly the analysis of additional somatic cell neighbourhoods will be required to address this issue more fully.

In summary, we demonstrate that in the case of the testis, the linear integrity of gene expression neighbourhoods, or the physical co-location in the nucleus of genes defining gene expression neighbourhoods in the *Drosophila* genome, is not required for normal gene expression. We conclude that models explaining the existence of neighbourhoods that rely on gene proximity or locality are unlikely to be sufficient to explain this conserved facet of genome organisation and suggest that more subtle effects, not easily detected under laboratory conditions, are selected by evolution to maintain neighbourhoods.

## Materials and Methods

### *Drosophila* Methods

All fly stocks were maintained on standard cornmeal-yeast-agar at 25°C. The RS element stocks used to generate the inversions are described in Table 1. Inversions were generated according to the crossing schemes described in Ryder et al. [43]. Salivary gland polytene chromosomes were prepared from EIN/+ larvae grown on yeast glucose food, stained with acetic acid-orcein, and viewed with a Zeiss Axiophot phase contrast microscope.

### Microarray Analysis

Adult male and female flies were separated at 4–7 d post-eclosion and aliquots of 12–15 flies transferred directly into 300 µl Trizol reagent. Total RNA was extracted according to our standard protocol and directly labelled by incorporation of Cy3 or Cy5 dCTP during first strand cDNA synthesis reactions. For testis samples, RNA was extracted from 4–7-d-old adult males and directly stored in Trizol. Eight pairs of testes devoid of accessory structures were pooled for RNA extraction and the samples labelled by random priming with Klenow polymerase after conversion to double-stranded cDNA. Embryos were aged between 0 and 21 h and dechorionated in bleach before being transferred directly into Trizol. Microarrays were printed in-house on PowerMatrix slides using a Qarray2 spotter with a set of long oligonucleotides (GEO platform accession GPL8244). After hybridisation and washing, microarrays were scanned at 5 µm resolution in a GenePix 4000B dual-laser scanner with GenePix Pro 5.1 imaging software using 100% laser power and individually optimised PMT gain settings. Spot-finding and signal quantification were performed with Dapple v0.88pre2 [77] followed by variance stabilizing normalisation [56]. Average expression values from biological replicates (a minimum of three), standard deviations, *t* statistics, and *p* values were calculated with Cyber T [78]. Full details of experimental protocols are available from www.flychip.org.uk. Over-representation of Gene Ontology terms was calculated using the Hypergeometric distribution and a Benjamini-Hochberg test correction in FlyMine (www.flymine.org) [79]. All of the raw microarray data are available from the NCBI Gene Expression Omnibus under series accessions GSE15565 and GSE21607.

### Real-Time PCR

Two µg total RNA was treated with 1U RQ1 DNase for 30 min at 37°C prior to reverse transcription. RNA was heated at 65°C for 10 min together with 500 ng anchored oligo(dT)23 primer and 10 nMoles dNTPs, briefly cooled on ice, and incubated with 1 µl RNAsin, 1 µMole DTT, 1x first strand buffer, and 200 U Superscript III Reverse Transcriptase for 1 h at 50°C. The reaction was terminated by incubation at 70°C for 15 min. cDNA synthesised from 8 ng total RNA was used as a template for quantitative real-time PCR. Real-time PCR was performed on the cDNA using the BioRad iQ5 Cycler Real-Time PCR Detection System, 2x SensiMix Plus SYBR and Fluorescein Kit (Quantace; Cat. No. QT615-02), 0.5 pMoles primer 1 and primer 2. Cycling was for 3 min at 95°C, followed by 60 cycles of 95°C for 10 s, 56°C for 30 s, and 77°C for 6 s. A melt curve was performed directly after the cycling to verify the products by increasing the temperature from 56°C to 95°C in 0.5°C increments and acquiring fluorescence data after each increment. Four independent samples for each genotype were assayed in three technical replicates. Expression for each gene was normalised to Rp49 using the deltaC_T_ method: Ratio (reference/target)  = 2^CT(reference) – CT(target)^, where reference  =  Rp49 and target  =  gene of interest. One-tailed *t* tests were performed to determine the significance of differences between the inversion and progenitor genotypes. See Table S6 for primer sequences used in qPCR.

### Probe Labelling for DNA FISH

Probes were designed to amplify genomic DNA regions with the neighbourhood, either side of the *In(2LR)EIN136* inversion breakpoint at 9510372. Primers were designed to amplify 10 different 2 kb products spread across ∼25 kb proximal region from 9479506 to 9503810 (C probes) and distal region from 9526878 to 9551429 (D probes). All 10 PCR products for each region were combined before labelling. Fluorescently labelled probes were generated by using the PCR products as the template for nick translation to enzymatically incorporate an amine-modified nucleotide into the probe template, followed by a second dye-coupling step. Probes were labelled either with Alexa Fluor 488 using FISH Tag DNA Green Kit (Invitrogen F32947) or Alexa Fluor 555 using FISH Tag DNA Orange Kit (Invitrogen F32948), according to the manufacturer's protocol. See Table S7 for primer sequences.

### Dissection, Fixation, and Hybridisation of Adult Testes

Testes were dissected from 1–2-d-old adult males from *In(2LR)EIN136* and its progenitor *cis* stock in PBS. Dissected testes were pooled in batches of 10 for each genotype and transferred to 1 ml fix (4% formaldehyde in PBT [PBS+0.1% Tween-20]) for 20 min, followed by 3×10 min washes in fresh PBT, and then continued directly with hybridisation steps. Two-colour DNA FISH on whole-mount tissues was performed as previously described [80], with minor modifications. See Protocol S1 for detailed protocol.

### Microscopy, 3D Image Analysis, and Statistical Analysis of the Data

Optimally spaced Z-stacks were collected using a Leica SP5 confocal microscope (Leica Microsystems) with a 63× 1.4NA λ-corrected objective. Measurement of distance between labelled sites was performed using Imaris software (Bitplane) with the “Measurement Points” function to measure the distance between pairs.

## Supporting Information

Figure S1 **Creating inversions.** Chromosomes containing an RS3 and RS5 element in *cis*, where one element resides within the gene expression neighbourhood, are created by recombination between two chromosomes carrying single elements. The *white* ^+^ RS elements are reduced by heatshock-induced FLP-recombinase to generate *w* ^−^ chromosomes. A second round of FLP recombinase treatment induces recombination between the two elements *in cis* generating an easily identified *w^+^* chromosome that is inverted between the *P* elements.
(0.30 MB JPG)

Figure S2 **Inversion cytology.** Confirmation of the inversions by cytological examination of salivary gland polytene chromosomes. For each of the indicated inversions, which are *in trans* with a wild type chromosome, the breakpoints are marked by the arrows and cytological locations are indicated.
(3.24 MB JPG)

Figure S3 **Neighbourhood 19F.** Genomic map of the 19F region from FlyBase with the indicated gene models. Above the map, the log2 expression ratios in the indicated genotypes. The triangle and arrow represents the location of the RS insertion. The lower graphs show the other ends of the inversion breakpoints with the gene expression measures.
(1.44 MB JPG)

Figure S4 **Neighbourhood 35F.** Genomic map of the 35F region from FlyBase with the indicated gene models. Above the map, the log2 expression ratios in the indicated genotypes. The triangle and arrow represents the location of the RS insertion. The lower graphs show the other ends of the inversion breakpoints with the gene expression measures.
(1.45 MB JPG)

Figure S5 **Neighbourhood 50B.** Genomic map of the 50B region from FlyBase with the indicated gene models. Above the map, the log2 expression ratios in the indicated genotypes. The triangle and arrow represent the location of the RS insertion. The lower graphs show the other ends of the inversion breakpoints with the gene expression measures.
(1.45 MB JPG)

Figure S6 **Embryo neighbourhood.** Heatmap of expression values for the 18E embryo domain (Spellman block 209). The log2 mean centred expression values for each gene in the neighbourhood across 88 experiments with RNA from embryo or adults is plotted according to the colour scale. Neighbourhood genes are indicated in bold. Data from Spellman and Rubin (2002), main text reference [14].
(0.70 MB JPG)

Figure S7 **Plots of mean signal to noise ratio of normalised A values in disrupted and intact neighbourhoods.** For each gene, the signal/noise ratio of vsn normalised A values was averaged for all samples within a replicate group (EIN and *cis* together or male and female together). Each gene within the 19F (A), 35F (B), or 50B (C) neighbourhood is represented by a different symbol. The distribution of signal/noise ratios does not differ between the experiments where a neighbourhood is disrupted (indicated by the black bars) and those in which it is intact.
(0.85 MB JPG)

Figure S8 **MA plots of average signal intensity versus log-ratio.** Genes within a neighbourhood are not significantly differentially expressed between inverted (EIN) and intact progenitor (*cis*) samples. M is the log differential expression ratio and A is the mean log intensity between the two channels. Small panels show normalised, log(2)-transformed data from individual slides of a replicate group and large panel shows the average values of the replicate group. Genes within the 35F and 50B neighbourhoods are shown in red, all other genes on the array are shown in black. Dotted green line shows loess fit.
(1.11 MB JPG)

Protocol S1 **Detailed DNA FISH protocol.**
(0.05 MB DOC)

Table S1 **Sex-specific expression.** For each of the three male-specific gene expression neighbourhoods, the FlyAtlas gene expression call (Up, enriched in testis; None, not enriched in testis; AG, enriched in male accessory gland), the average log2 expression ratio and *p* value for male versus female comparisons in *In(2L)EIN133/In(2L)EIN133* and *EIN133-cis/EIN133-cis*.
(0.00 MB TXT)

Table S2 **Neighbourhood gene expression measures.** Average log2 expression ratios and *p* values for the indicated genotypes for each of the four neighbourhoods tested.
(0.01 MB TXT)

Table S3 **Complete dataset for 19F, 35F, and 50B inversions.** UniqueID, spot identifier; Clone-ID, FlyBase transcript identifier; FlyBaseID, unique FlyBase identifier; FlyBase_gene, gene name. For each indicated genotype the results of the CyberT analysis are provided: number of arrays (N), average log2 expression ratio (x), standard deviation (sd), *t* statistic (t), and *p* value (p).
(9.48 MB TXT)

Table S4 **Complete dataset for 18E inversion in embryos.** UniqueID, spot identifier; Clone-ID, FlyBase transcript identifier; FlyBaseID, unique FlyBase identifier; FlyBase_gene, gene name. For each indicated genotype the results of the CyberT analysis are provided: number of arrays (N), average log2 expression ratio (x), standard deviation (sd), *t* statistic (t), and *p* value (p).
(1.49 MB TXT)

Table S5 **Significant gene expression changes.** For each inversion the average log 2 ratio and *p* values for genes with significant expression changes are listed (>1.5-fold change, *p*<0.01).
(0.01 MB TXT)

Table S6 **Primer sequences for RealTime PCR probes.** The cytological location and sequence of PCR primer pairs and product size for RealTime PCR are listed.
(0.00 MB TXT)

Table S7 **Primer sequences for DNA FISH probes.** The cytological location and sequence of PCR primer pairs for generating DNA FISH probes are listed.
(0.00 MB TXT)

Table S8 **Signal to noise levels.** For each gene within the three testis neighbourhoods, the average VSN normalised intensity value for each replica group (Average A), the standard deviation of this measure (SD), and the signal to noise ratio were calculated. The Spot status (A, accept; R, reject; S, suspect) assigned by the Dapple spotfinder is also indicated. Generally the signal to noise measures are very high and similar irrespective of whether or not the neighbourhood is disrupted. Low S/N ratios are observed where suspect or rejected spots are identified.
(0.03 MB TXT)

Table S9 **Expression ratio confidence values.** 90% and 95% confidence intervals for the average expression ratio (M value) of each gene in each experiment. The Spot status (A, accept; R, reject; S, suspect) assigned by the Dapple spotfinder is also indicated.
(0.04 MB TXT)
