# Supplementary figures and images for: Neighbourhood Continuity Is Not Required for Correct Testis Gene Expression in Drosophila
Source: PLoS Biol. 2010 Nov 30;8(11):e1000552. doi: 10.1371/journal.pbio.1000552 (PMC2994658; doi:10.1371/journal.pbio.1000552)

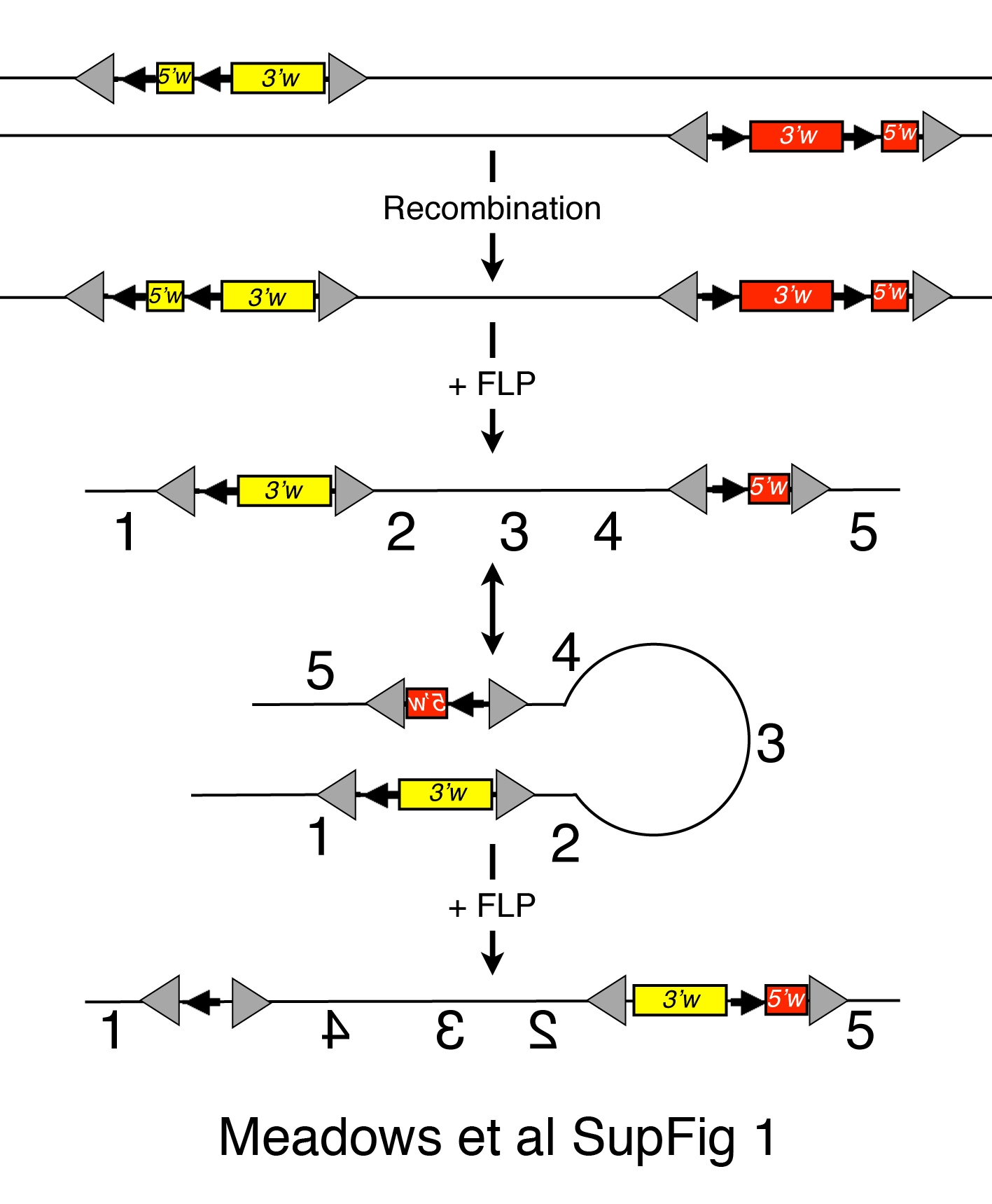

Supplement: Figure S1 — Creating inversions. Chromosomes containing an RS3 and RS5 element in cis, where one element resides within the gene expression neighbourhood, are created by recombination between two chromosomes carrying single elements. The white + RS elements are reduced by heatshock-induced FLP-recombinase to generate w − chromosomes. A second round of FLP recombinase treatment induces recombination between the two elements in cis generating an easily identified w+ chromosome that is inverted between the P elements. (0.30 MB JPG) [file pbio.1000552.s001.jpg]

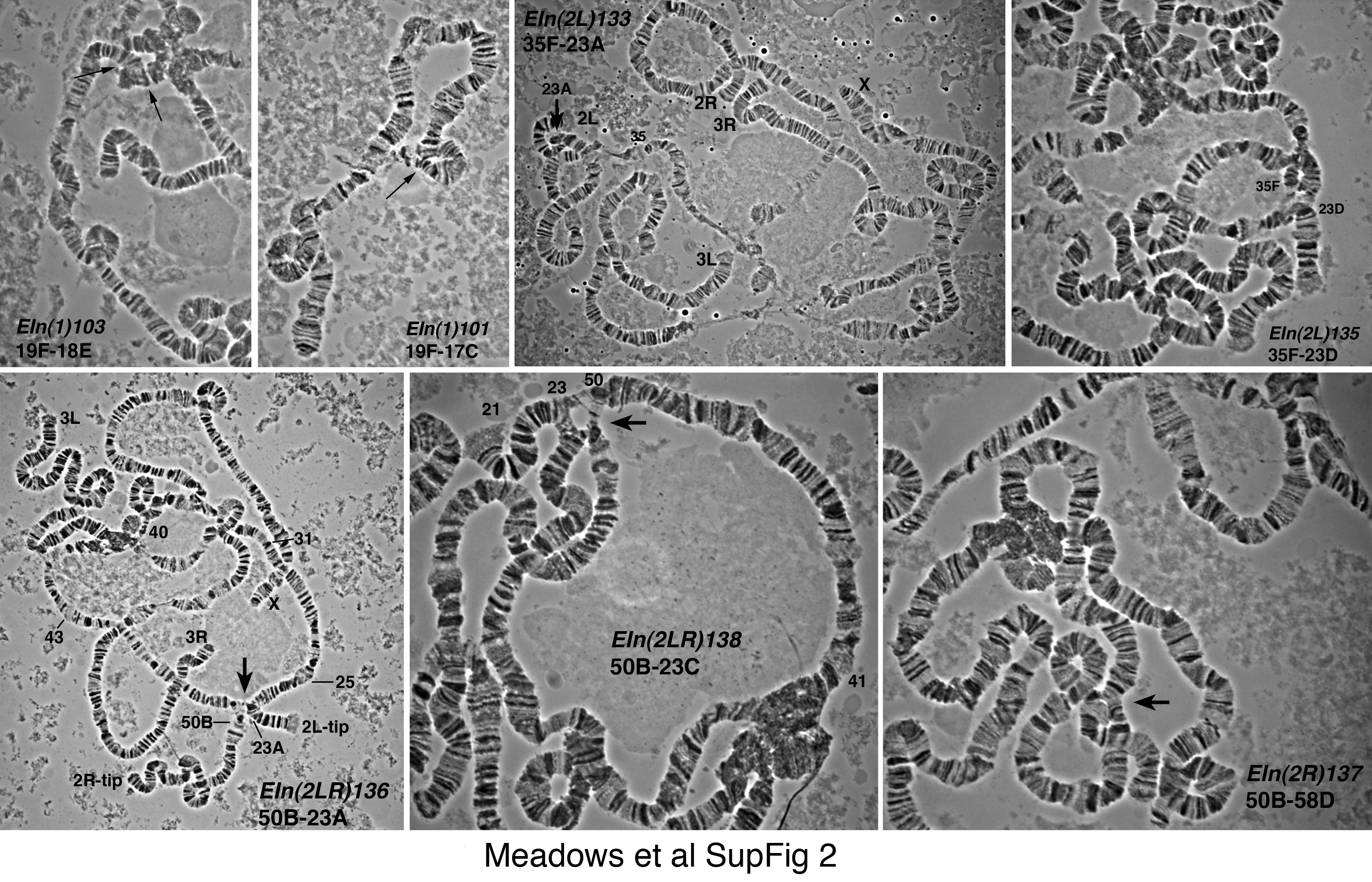

Supplement: Figure S2 — Inversion cytology. Confirmation of the inversions by cytological examination of salivary gland polytene chromosomes. For each of the indicated inversions, which are in trans with a wild type chromosome, the breakpoints are marked by the arrows and cytological locations are indicated. (3.24 MB JPG) [file pbio.1000552.s002.jpg]

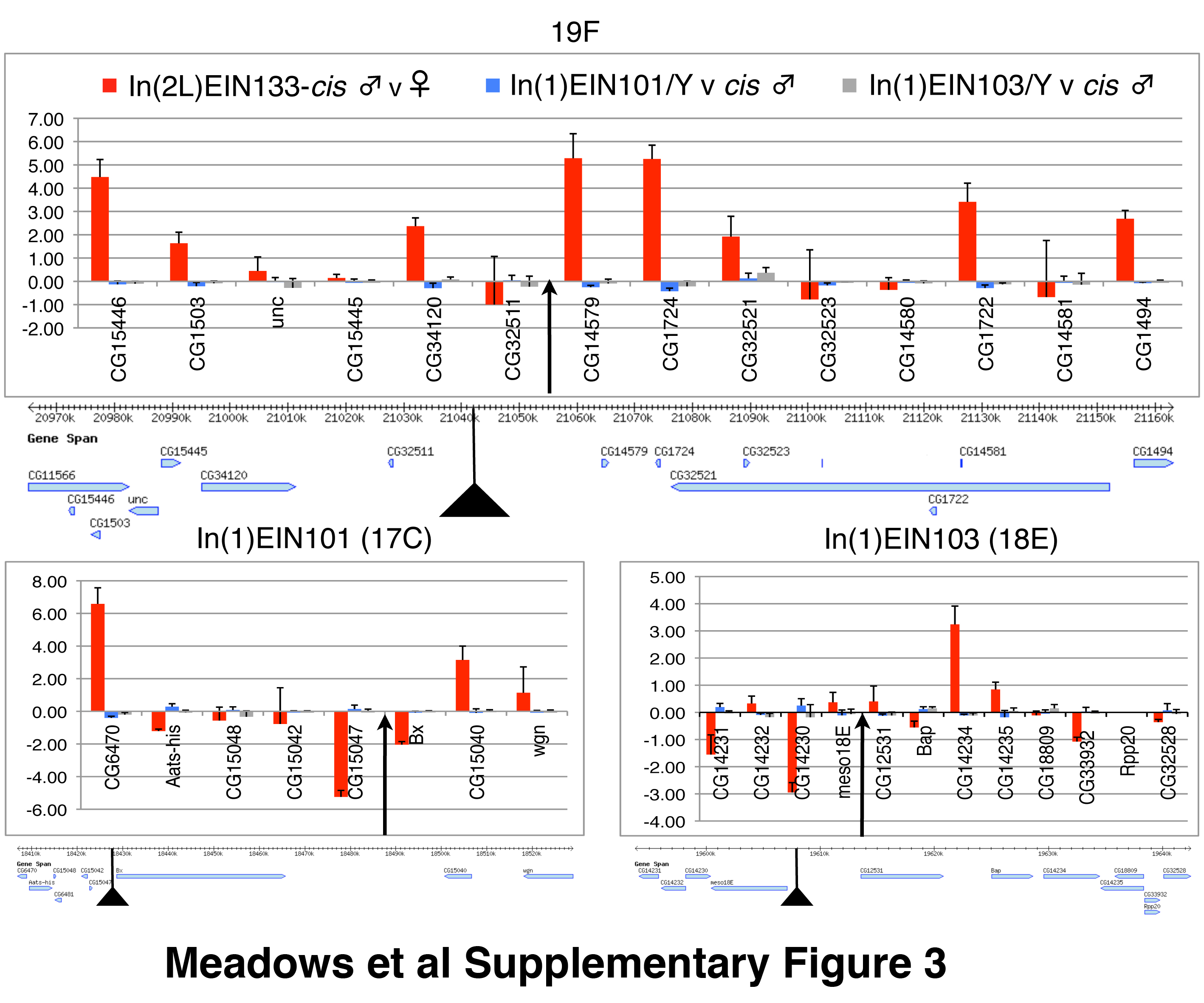

Supplement: Figure S3 — Neighbourhood 19F. Genomic map of the 19F region from FlyBase with the indicated gene models. Above the map, the log2 expression ratios in the indicated genotypes. The triangle and arrow represents the location of the RS insertion. The lower graphs show the other ends of the inversion breakpoints with the gene expression measures. (1.44 MB JPG) [file pbio.1000552.s003.jpg]

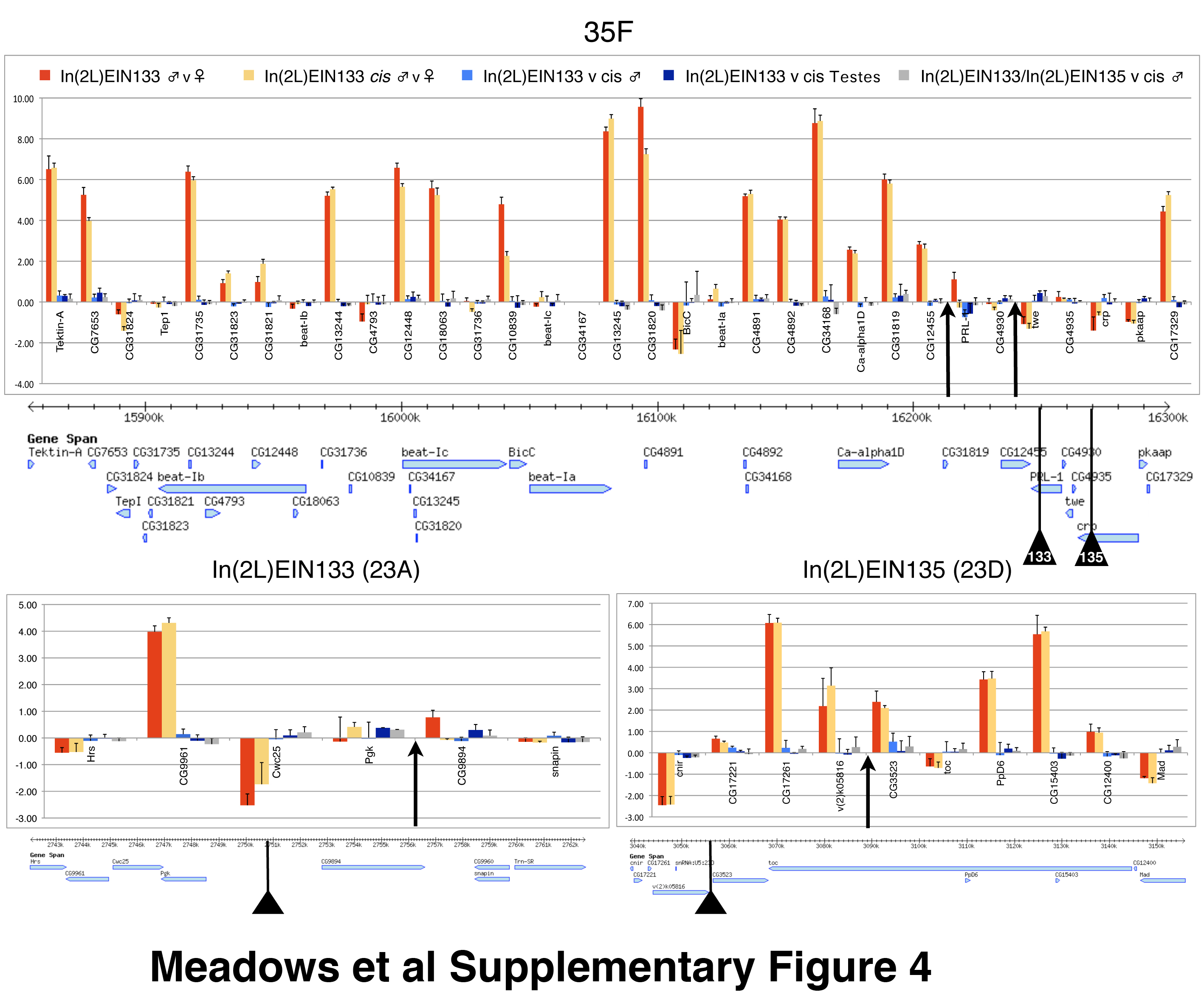

Supplement: Figure S4 — Neighbourhood 35F. Genomic map of the 35F region from FlyBase with the indicated gene models. Above the map, the log2 expression ratios in the indicated genotypes. The triangle and arrow represents the location of the RS insertion. The lower graphs show the other ends of the inversion breakpoints with the gene expression measures. (1.45 MB JPG) [file pbio.1000552.s004.jpg]

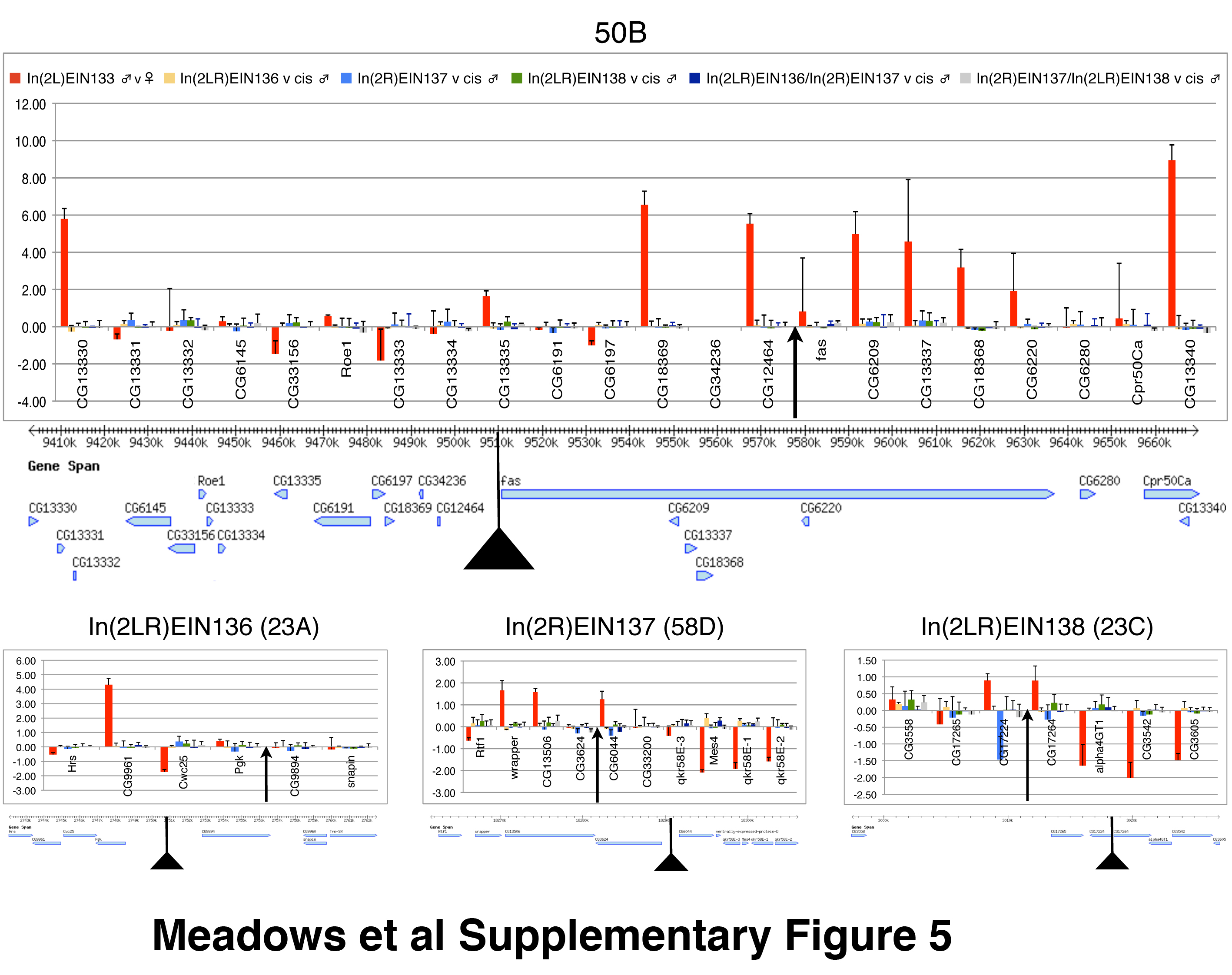

Supplement: Figure S5 — Neighbourhood 50B. Genomic map of the 50B region from FlyBase with the indicated gene models. Above the map, the log2 expression ratios in the indicated genotypes. The triangle and arrow represent the location of the RS insertion. The lower graphs show the other ends of the inversion breakpoints with the gene expression measures. (1.45 MB JPG) [file pbio.1000552.s005.jpg]

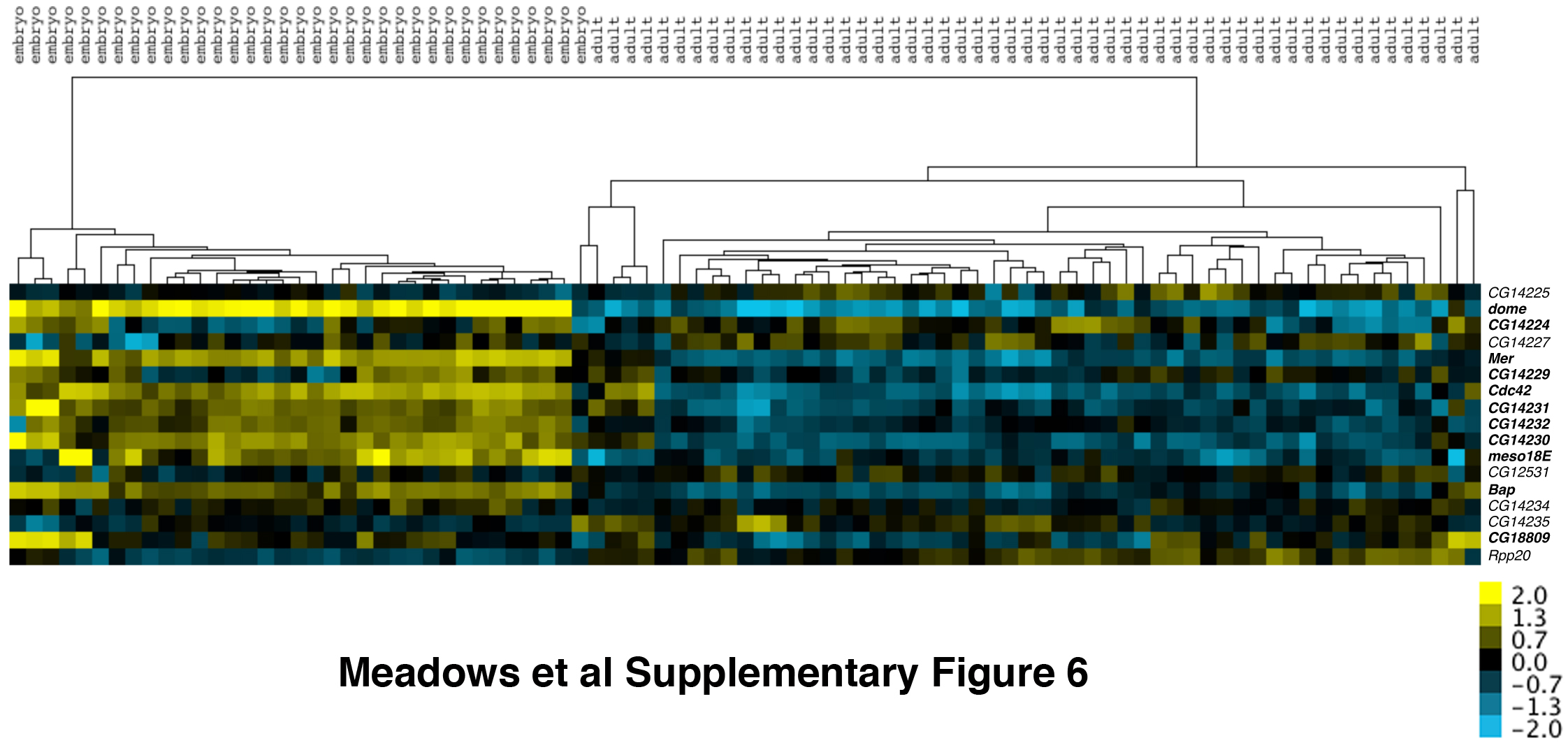

Supplement: Figure S6 — Embryo neighbourhood. Heatmap of expression values for the 18E embryo domain (Spellman block 209). The log2 mean centred expression values for each gene in the neighbourhood across 88 experiments with RNA from embryo or adults is plotted according to the colour scale. Neighbourhood genes are indicated in bold. Data from Spellman and Rubin (2002), main text reference [14]. (0.70 MB JPG) [file pbio.1000552.s006.jpg]

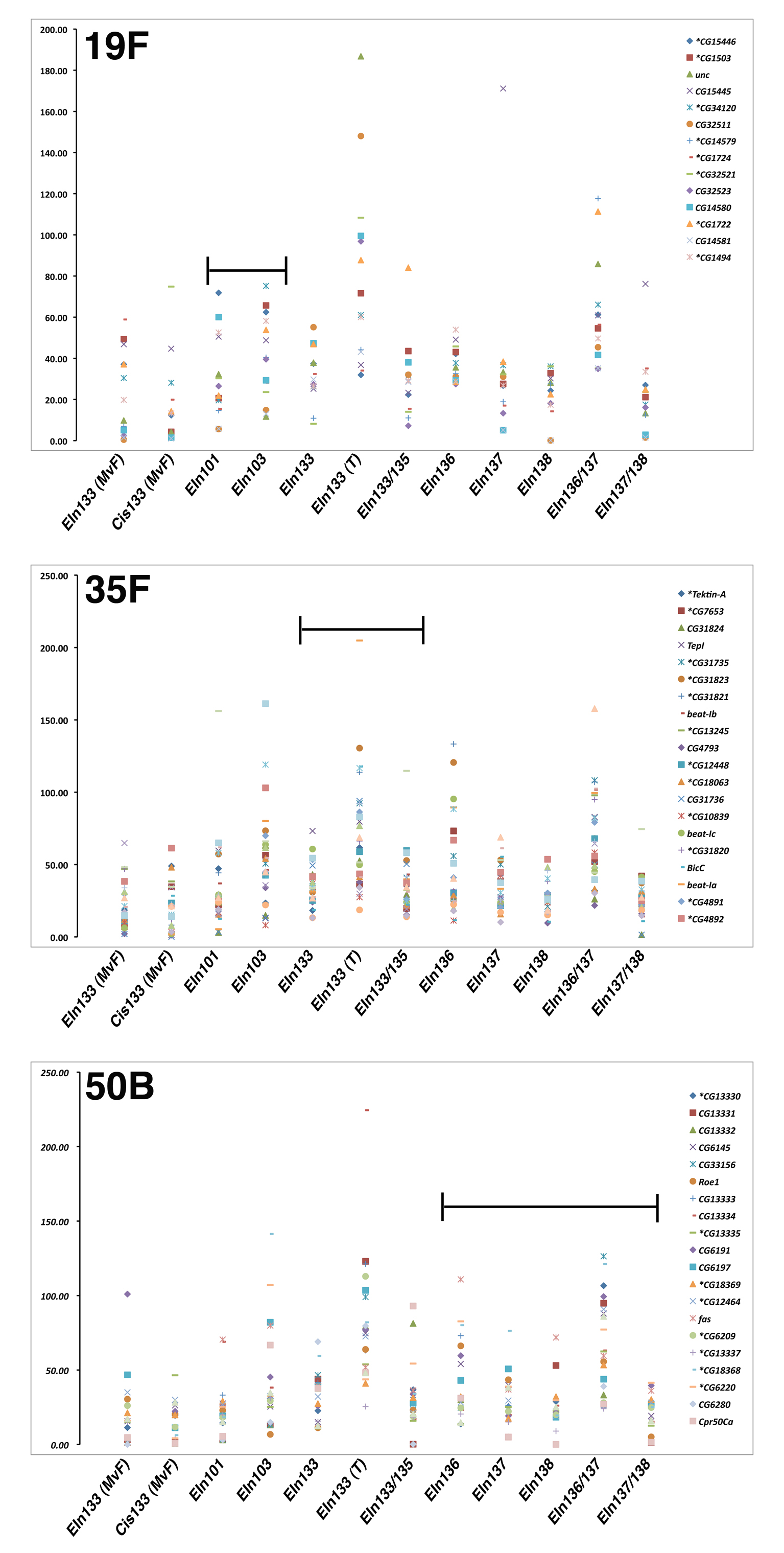

Supplement: Figure S7 — Plots of mean signal to noise ratio of normalised A values in disrupted and intact neighbourhoods. For each gene, the signal/noise ratio of vsn normalised A values was averaged for all samples within a replicate group (EIN and cis together or male and female together). Each gene within the 19F (A), 35F (B), or 50B (C) neighbourhood is represented by a different symbol. The distribution of signal/noise ratios does not differ between the experiments where a neighbourhood is disrupted (indicated by the black bars) and those in which it is intact. (0.85 MB JPG) [file pbio.1000552.s007.jpg]

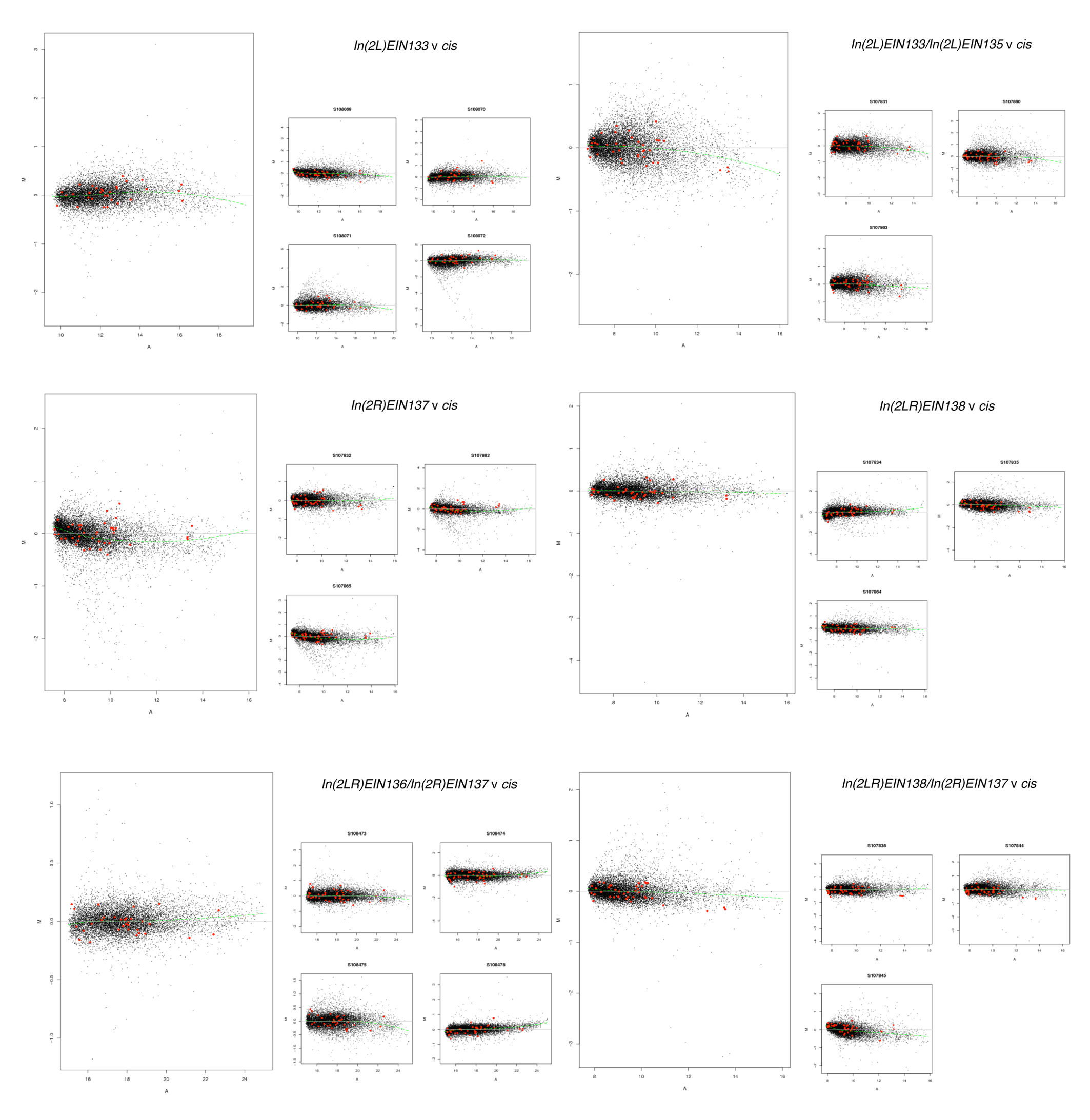

Supplement: Figure S8 — MA plots of average signal intensity versus log-ratio. Genes within a neighbourhood are not significantly differentially expressed between inverted (EIN) and intact progenitor (cis) samples. M is the log differential expression ratio and A is the mean log intensity between the two channels. Small panels show normalised, log(2)-transformed data from individual slides of a replicate group and large panel shows the average values of the replicate group. Genes within the 35F and 50B neighbourhoods are shown in red, all other genes on the array are shown in black. Dotted green line shows loess fit. (1.11 MB JPG) [file pbio.1000552.s008.jpg]
