# Supplementary material for: Neighbourhood Continuity Is Not Required for Correct Testis Gene Expression in Drosophila
Source: PLoS Biol. 2010 Nov 30;8(11):e1000552. doi: 10.1371/journal.pbio.1000552 (PMC2994658; doi:10.1371/journal.pbio.1000552)
Supplement: Protocol S1 — Detailed DNA FISH protocol. (0.05 MB DOC) [file pbio.1000552.s009.doc]

**DNA FISH on testes: 24 well plates/1.5ml tubes (no proteinase K step)**

DNA-FISH-Tag probes **updated 17/9/09**

**Fixation of testes**

1. Dissect the testes in autoclaved PBS1X. Transfer into a mesh in a 24-well plate in 1ml PBS.
2. Once there are 10 testes pairs, transfer mesh to next well containing fix:

PBT (PBS 1X: 0.1% Tween20), 4% formaldehyde for 20 min

For 1ml **fix**:

Formaldehyde stock (40%) 100ul

10x PBS 100ul

10% Tween-20 10ul

H2O 790ul

Whilst fixing, continue with dissection of the next sample.

1. Wash the testes after fixation, 3 x 5-10 min in PBT

For 100ml **PBT** (=1xPBS, 0.1% Tween-20):

10x PBS 10ml

10% Tween-20 1ml

DEPC H2O 89ml

Leave in the last wash until ready to proceed with all samples.

Continue with subsequent steps by processing all the samples simultaneously.

**In situ DNA hybridization to fixed testes**

The following steps are performed in meshes in 24 well plates with 1ml liquid in at RT. Gentle agitation (eg. 50rpm) can be used.

**Pre-Hybridization**

1. After fixation, incubate testes in 1 ml of 100-200 ug/ml RNAse A in PBT for at least 2 hours at RT.

RNAse A 100x stock (10mg/ml) 10ul

PBT 990ul

1. Incubate testes for at least 1 hour at RT in 1ml of PBS-Tr (PBS 1X, 0.3% TX-100).

For 100ml PBS-Tr (PBS + 0.3% Triton X-100);

10x PBS 97ml

10% Triton X-100 3ml

1. Transfer tissues into a pre-Hybridization Mixture (pHM) by passing though the following solutions (1ml each, 20 min each step):
2. 80% PBS-Tr; 20% pHM (0.8ml + 0.2ml)

***96 degree** **heating block on, 80 degree waterbath on – put foil on top***

9. 50% PBS-Tr; 50% pHM (0.5ml + 0.5ml)

10. 20% PBS-Tr; 80% pHM (0.2ml + 0.8ml)

***Set hotblocks to 80C and 95C***

11. 100% pHM. Transfer testes from meshes into 1.5ml tubes

***prepare probe and control FHB***

# Denaturation

12. Denature testis DNA by incubation for 15 min at 80°C in 100% pHM

13. Meanwhile, denature the DNA-FISH-Tag probes in FHB by incubating for 10 min at 95°C. Remove as much liquid from the testes as possible, then immediately add the probes to the testes (without prior cooling). Use 150-200 ng DNA in about 30ul of FHB/hybridization.

SUBSEQUENT STEPS SHOULD BE PERFORMED IN THE DARK

**Hybridization**

14. Incubate for 14-17hrs at 37C (in waterbath).

**Post-hybridization washes**

***Put 45 degree and 37 degree waterbaths on***

15. Transfer testes back into meshes.

(Preheat wash solutions in 45/37°C waterbath. Place 24 well plate containing meshes in waterbath. Rest on a rack so that the bottom of the plate is just above the water).

16. Wash 1ml solution 1 for 10mins at 45°C

17. Transfer to 1ml fresh solution 1 for 20mins at 37°C

18. Continue with post-hyb washes 2, 3 and 4 at 37°C and 5-7 at RT (1ml each, 20mins each step).

*Cavalli lab generally performs the first wash for 10 minutes at 45°C followed by solution 1 again at 37°C to improve stringency.

Wash the tissues by adding post-hybridization wash solutions 1 to 7 (40ml each, 20 min each step)**.** Washes 1- 4 in 37°C incubator, 5-7 at RT.

**Post-hybridization wash solutions (make them fresh)**

**1** 50% formamide (Fluka) ; 2X SSC ; 0.3% CHAPS @37°C

100% formamide 50ml

20x SSC 10ml

10% CHAPS 3ml

DEPC H2O 37ml

**2** 40% formamide ; 2X SSC ; 0.3% CHAPS @37°C

100% formamide 40ml

20x SSC 10ml

10% CHAPS 3ml

DEPC H2O 47ml

**3** 30% formamide ; 70% PBT @37°C

100% formamide 30ml

PBT 70ml

**4** 20% formamide ; 80% PBT @37°C

100% formamide 20ml

PBT 80ml

Subsequent washes at RT:

**5** 10% formamide ; 90% PBT

100% formamide 10ml

PBT 90ml

**6** 100% PBT

**7** 100% PBS-Tr

**DAPI stain and mounting**

19. Wash in PBT + 100ng/ml DAPI (1 in 10 dilution of 1ug/ml stock). 20mins RT.

20. Rinse quickly in PBS (in order to remove all traces of detergent).

21. Transfer testes from meshes to slide in a small volume of liquid with a cut-off pipette tip.**

22. Blot off as much liquid as possible

23. Add 40ul ProLong Gold antifade.

24. Place 22x22mm coverslip on top, avoiding bubbles.

25. Allow to harden and store at 4°C at least one night at 4°C before acquisitions. Mounted tissues in ProLong antifade can be stored for up to 3 weeks at 4°C.

**NB. Had problems with testes sticking to the pipette when transferring from mesh to slide. Next time try adding antifade first and then transferring to slide.

------------------------------------------------------------------------------------------------------

**Materials & Reagents**

**PHM pre-Hybridization Mixture (make it fresh):**

50% formamide (Fluka) (breaks up hybrids)

4X SSC

100 mM NaH2PO4, pH 7.0

0.1% Tween 20

For 100ml PHM:

100% formamide 50ml

20x SSC 20ml

1M NaH2PO4, pH7.0 10ml

10% Tween 20 1ml

DEPC H2O 19ml

**Post-hybridization wash solutions (make them fresh)**

**1** 50% formamide (Fluka) ; 2X SSC ; 0.3% CHAPS

100% formamide 50ml

20x SSC 10ml

10% CHAPS 3ml

DEPC H2O 37ml

**2** 40% formamide ; 2X SSC ; 0.3% CHAPS

100% formamide 40ml

20x SSC 10ml

10% CHAPS 3ml

DEPC H2O 47ml

**3** 30% formamide ; 70% PBT

100% formamide 30ml

PBT 70ml

**4** 20% formamide ; 80% PBT

100% formamide 20ml

PBT 80ml

**5** 10% formamide ; 90% PBT

100% formamide 10ml

PBT 90ml

**6** 100% PBT

**7** 100% PBS-Tr

**FISH Hybridization Buffer (can be stored at -20°C for long time storage, and pre-warmed at 37°C before use):**

2X SSC

10% dextransulfat (Pharmacia) (effectively increases probe concentration by taking up volume)

50% deionized formamide (Sigma)

Salmon Sperm DNA at 0.5 mg/ml (0.05% final, i.e. 2% of a 2.5% sonicated and autoclaved stock solution)

For 10ml **FISH hyb buffer**:

20x SSC 1ml

50% dextran sulphate 2ml

100% deionised formamide 5ml

10mg/ml sssDNA 0.5ml

DEPC H2O 1.5ml

**General reagents**

BD Falcon Cell Culture Inserts, 8 micron pore size, PET membrane (BD Biosciences, cat. No. 353097)

Bidistilled H_2_O (ddH_2_O)

10X PBS, pH 7.4 (Gibco)

PIPES (Sigma)

NaH_2_PO_4_ (Prolabo)

20X SSC (Euromedex)

Triton X-100 (USB)

Tween 20 (USB)

CHAPS (3-[(3-cholamidopropyl) dimethylammonio]-1-propanesulfonate) (Sigma)

**Kits and reagents for probe labeling**

FISH Tag DNA green kit (Alexa 488 dye) – Invitrogen cat # F32947

FISH Tag DNA orange kit (Alexa 555 dye) – Invitrogen cat # F32948

**Solutions for FISH hybridization**

PBT (PBS 1X; 0.1% Tween20)

PBS-Tr (PBS 1X; 0.3% Triton X-100)

RNAse A (Sigma R-6513); stock solution 10mg/ml; 100X)

Dextransulfat Mw 500,000 (Pharmacia 17-0340-01): to make a 50% solution, use the recipe of Pharmacia: weigh 5g of Dextransulfat in a 50ml Falcon tube, add 7ml of pre-warmed ddH_2_O at 60°C, and agitate for at least 30 minutes using a magnetic stirrer

Formamide (Fluka) and deionized formamide (Sigma F-9037)

Mineral oil (Sigma)

DAPI (4'6' Diamidino-2-phenylindole).

**Mounting Media**

Prolong Gold antifade (Molecular Probes)
